# Assessing the effect of soil cultivation methods and genotypes on crop yield components, yield and soil properties in wheat (*Triticum aestivum* L.) and Rice (*Oryza sativa* L.) cropping system

**DOI:** 10.1186/s12870-024-05001-y

**Published:** 2024-04-29

**Authors:** Ankit Saini, Sandeep Manuja, Ram Gopal Upadhyay, Shilpa Manhas, Chinmaya Sahoo, Gurudev Singh, Raj Paul Sharma, Riya Johnson, Joy M. Joel, Jos T. Puthur, Muhammad Imran, Mohammad Reza Fayezizadeh

**Affiliations:** 1 Department of Agronomy, Dr. Khem Singh Gill Akal College of Agriculture, Eternal University, Baru Sahib, Sirmaur, HP 173101 India; 2 Department of Agronomy, College of Agriculture, CSKHPKV, Palampur, HP 176062 India; 3 Department of Organic Agriculture and Natural farming, College of Agriculture CSKHPKV, Palampur, HP 176062 India; 4 Department of Agronomy, Lovely Professional University, Phagwara, Punjab 144411 India; 5 Department of Agronomy, College of Agriculture, Kerala Agricultural University, Vellayani, Thrissur, 680656 India; 6 Department of Soil Science, College of Agriculture CSKHPKV, Palampur, HP 176062 India; 7 Plant Physiology and Biochemistry Division, Department of Botany, University of Calicut, C.U. Campus P.O., Kerala, 673635 India; 8 Department of Soil and Environmental Sciences, MNS-University of Agriculture, Multan, 60000 Pakistan; 9 Department of Horticultural Science, Faculty of Agriculture, Shahid Chamran University of Ahvaz, Ahvaz, 61357-43311 Iran

**Keywords:** Conventional tillage, Natural farming, Nutrient content, Rice, Wheat and yield

## Abstract

**Background:**

The rice-wheat cropping system is the prevailing agricultural method in the North-Western states of India, namely in the Indo-Gangetic plains. The practice of open burning of rice residue is frequently employed for expedient land preparation, but it has significant adverse impacts on both the environment and human health. These include the emission of greenhouse gases, loss of nutrients, elevated concentrations of particulate matter (PM), and disruption of the biological cycle. This research aims to investigate the implementation of effective management strategies in the rice-wheat cropping system, namely via the use of tillage-based crop cultivation techniques, stubble retention, and integration approaches. The objective is to enhance soil health features in order to augment crop yield and improve its attributes.

**Results:**

The research was carried out using a split plot experimental design, consisting of three replications. The main plot consisted of four different cultivation methods, while the subplot included three genotypes of both rice and wheat. The research demonstrates the enhanced efficacy of residue application is significantly augmenting soil nutrient concentrations compared to standard tillage practices (*P* < 0.05). This was accomplished by an analysis of soil nutrient levels, namely nitrogen (N), phosphorus (P), potassium (K), and organic carbon (OC), at a depth of 0–15 cm. The implementation of natural farming, zero tillage, and reduced tillage practices resulted in decreases in rice grain yields of 34.0%, 16.1%, and 10.8%, respectively, as compared to conventional tillage methods. Similarly, the implementation of natural farming, zero tillage, and reduced tillage resulted in reductions in wheat grain yields of 59.4%, 10.9%, and 4.6% respectively, in comparison to conventional tillage practices.

**Conclusion:**

Regarding the individual crop genotypes investigated, it was continuously observed that Him Palam Lal Dhan 1 and HPW 368 displayed considerably greater grain yields for both rice and wheat during the two-year experimental period. Furthermore, when considering different cultivation methods, conventional tillage emerged as the most effective approach for obtaining higher productivity in both rice and wheat. Additionally, Him Palam Lal Dhan 1 and HPW 368 exhibited superior performance in terms of various crucial yield components for rice (such as panicle density, grains per panicle, panicle weight, and test weight) and wheat (including effective tiller density, grains per spike, spike weight, and 1000-grain weight).

## Background

Rice-wheat cropping system is among the world’s largest agricultural production systems and a major contributor to food security in South Asia [1, 2]. The rice-wheat cropping system covers 12.3 million hectares in India [3], generating about one-third of the region’s rice and wheat to nourish 15% of the population worldwide [4]. In India, rice is cultivated in an area of 43.38 million ha with a production of 130.29 million t and average productivity of 2809 kg ha^− 1^, whereas the comparable statistics for wheat are 30.47 million ha, 106.84 million t and 3507 kg ha^− 1^, respectively [5]. The increased production of rice and wheat in the various states of India is a consequence of excessive exploitation of natural resources like groundwater, soil, and energy [6]. However, in the state of Himachal Pradesh, these crops are grown under rainfed conditions with limited resources.

Post-green revolution, the use and dependence on chemical fertilizers for enhancing crop yield have significantly increased in Indian agriculture [7]. The green revolution undoubtedly contributed to the increase in agricultural production leading to the self-sufficiency in food grains [8, 9]. It also encouraged the use of chemical fertilizers in conjunction with high-yielding, nutrient and water-responsive varieties [10]. Due to stagnation in output levels, many have questioned the sustainability of the system. The productivity of the rice-wheat system is threatened by a plethora of factors, such as a dropping groundwater table, groundwater salinity, deteriorating soil health, increased weed incidence, diseases and pests [3]. The differing nutritional needs for rice and wheat can be considered one of the primary edaphic causes of the yield drop [11]. A puddled situation that destroys the soil structure is necessary for a successful rice harvest [12, 13]. Contrarily, wheat thrives in well-drained, well-formed soil [14, 15]. The hardpan that has developed hinders the development of the wheat’s roots, limits aeration, and lessens drainage. Since submerged soils frequently experience pH changes, this disrupts the chemical equilibria, which in turn affects the availability of various plant nutrients [16]. Due to the population of South Asia’s need for staple foods, the rice-wheat system has been and will continue to be the dominant system and has to be addressed to remain sustainable.

The traditional tillage techniques are simple to use and maintain a clean crop area. However, these practices involve excessive use of fuel and energy, particularly in the rice-wheat cropping system which is predominant in Indo-Gangetic plains. The adoption of conservation tillage practices like ridge-till, minimal tillage, and no-till tillage helps in reducing this excessive use of fuel and energy leading to sustainable crop production [17]. Ridge-till, minimal tillage, and no-till tillage techniques all leave residues on the soil surface and provide better erosion management. Conservation agriculture aims to boost agricultural production while simultaneously offering benefits to the economy and environment. “Future agriculture” is the name given to it [18]. A minimum amount of soil disturbance, an appropriate organic soil cover using residues or leguminous cover, as well as measures taken to lessen soil compaction through controlled traffic, are all crucial elements. In addition, plant leftovers utilized as mulch assist in feeding plants with nutrients when they decompose through the action of microorganisms. Therefore, it’s crucial to develop technologies that might provide higher yields with fewer resources, reduce tillage expenses, and boost farmers’ profit margins [19, 20].

A number of high-yielding, disease-resistant rice and wheat genotypes have been developed by breeders all over. However, most of these genotypes and their agro-techniques have been developed for use under conventional tillage. The performance of rice and wheat genotypes can vary under different growth conditions. Changes in the micro-climate of the field brought about by different tillage methods may have a significant impact on the performance of rice and wheat genotypes [21, 22]. Though some work on this aspect has been done at a global level with certain genotypes recommended for conservation tillage [23, 24], very little work has been done in India. Keeping this in mind the present investigation was carried out to assess the effect of cultivation methods and crop genotypes on the productivity and nutrient content of rice-wheat cropping system.

## Methods

### Experimental site and weather conditions

**Fig. 1:**
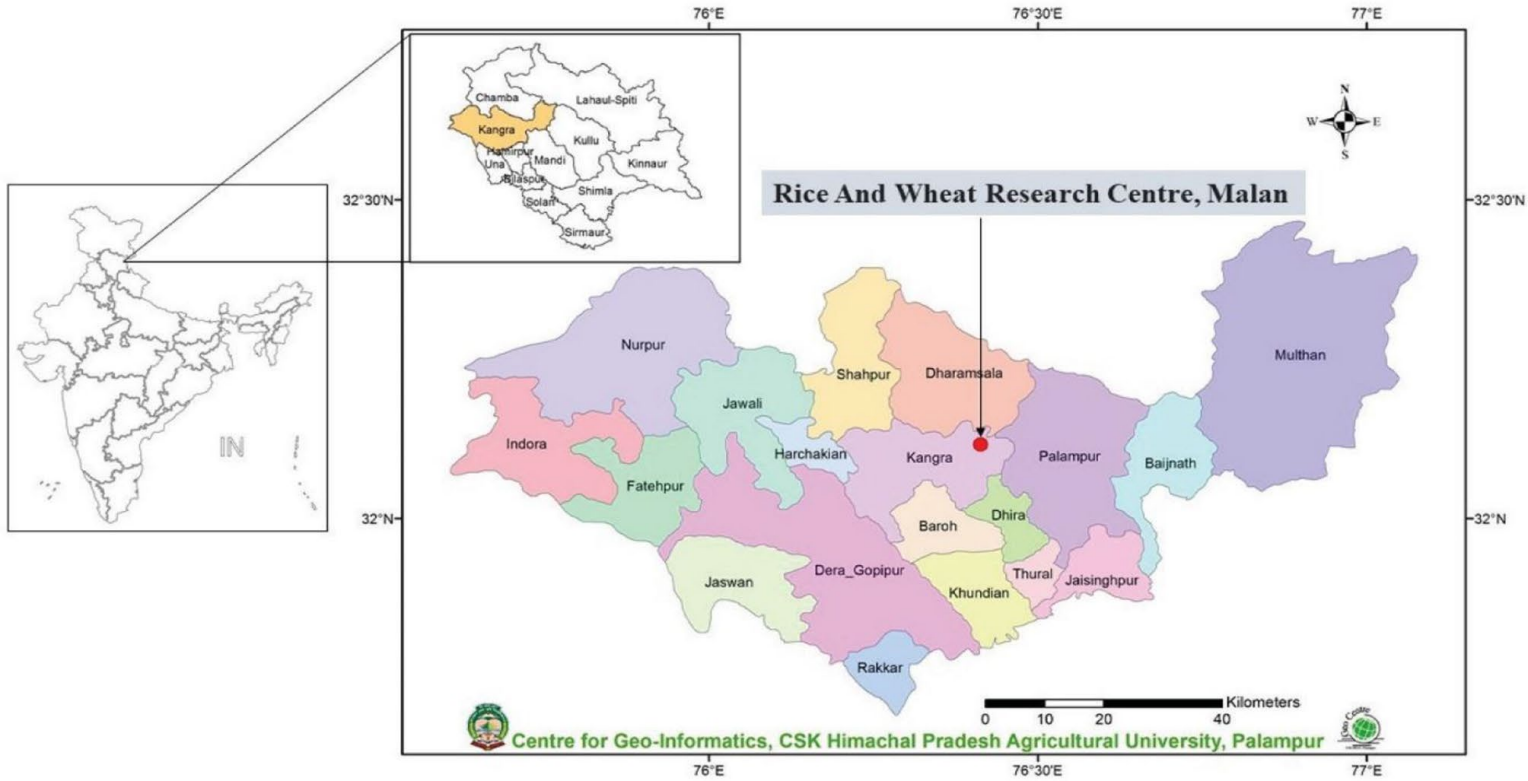
Geographical location of experimental site

**Fig. 2:**
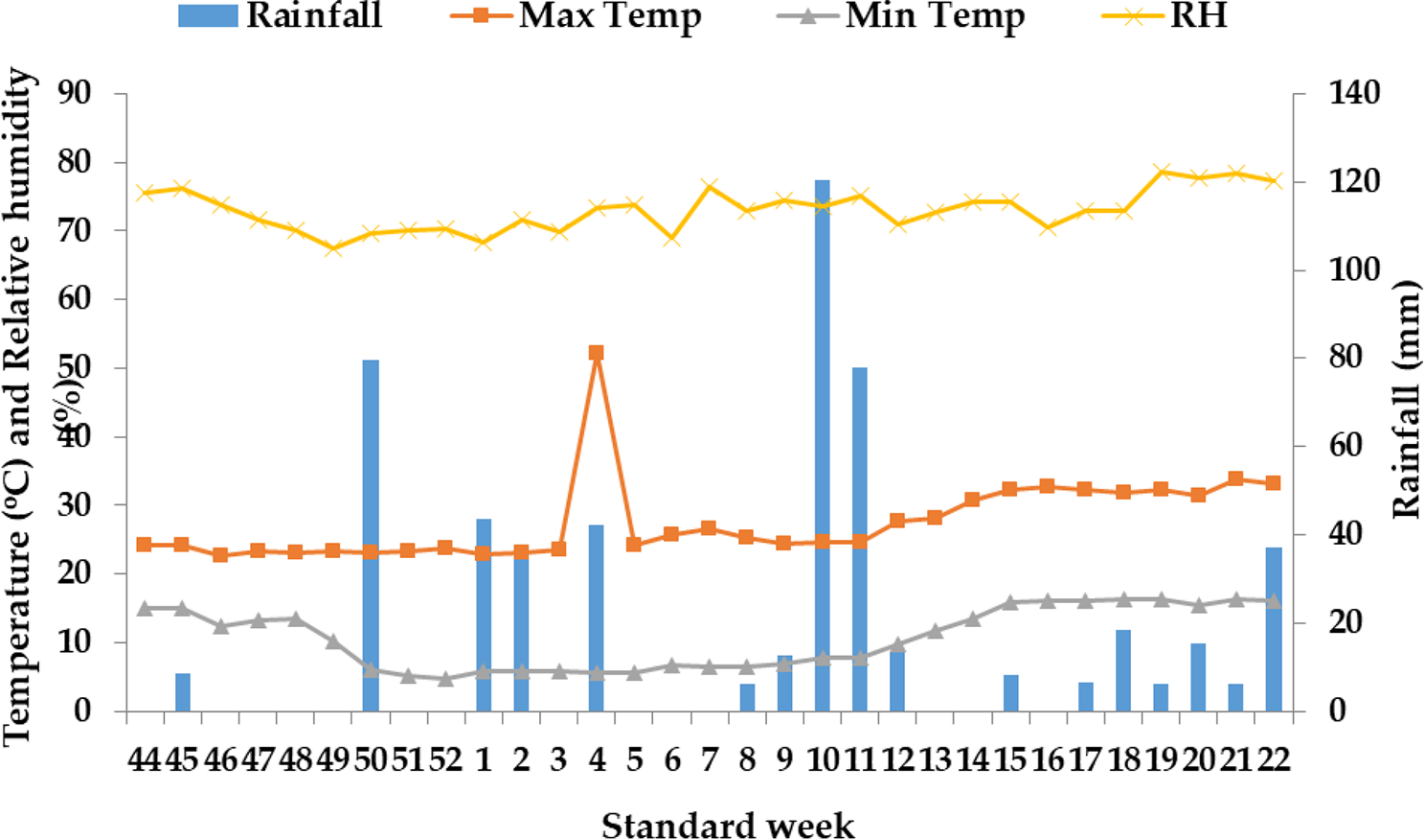
Mean weekly meteorological data at Malan during November 2019 to May 2020

**Fig. 3:**
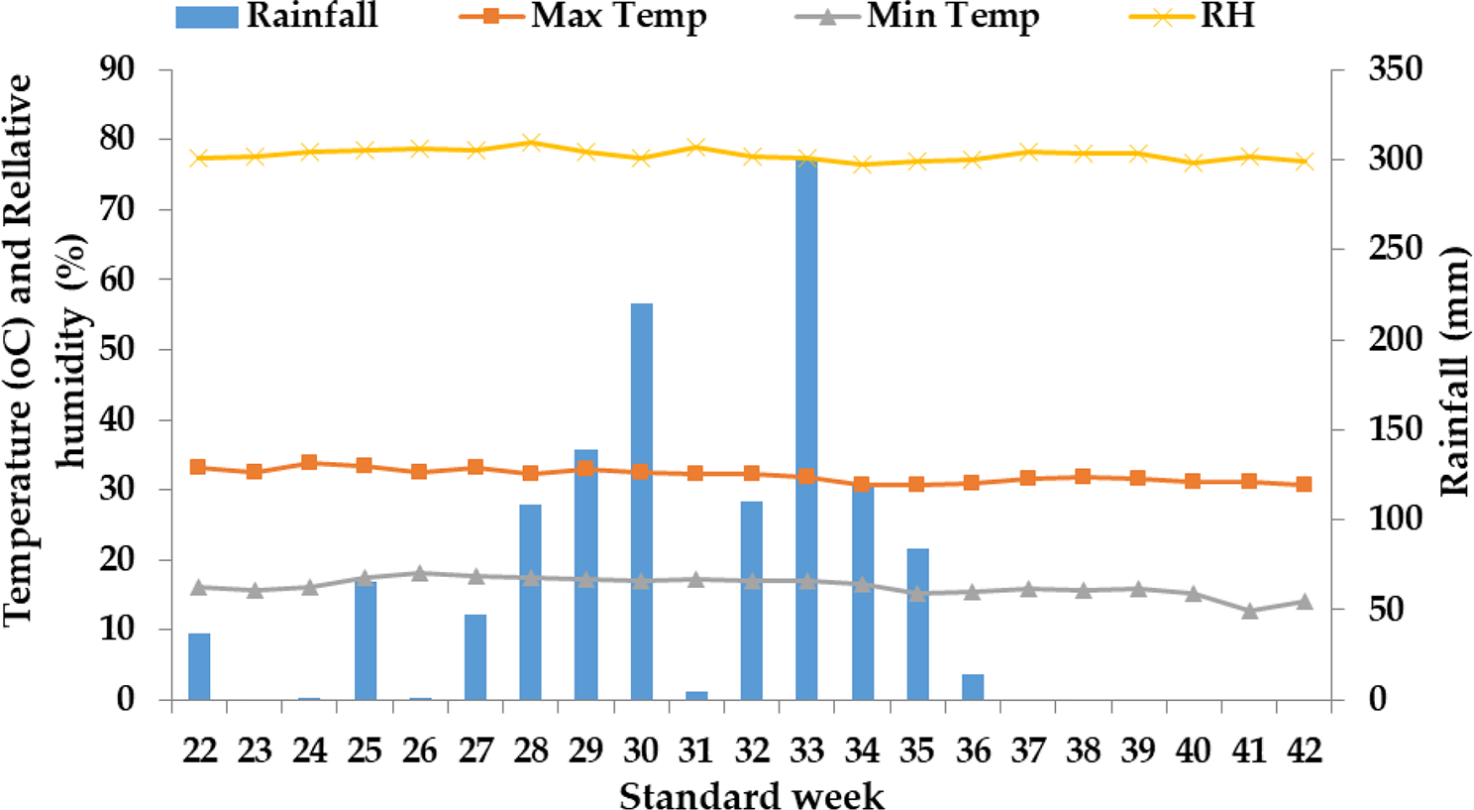
Mean weekly meteorological data at Malan during November 2020 to May 2021

A two-factor field investigation spanning three years from 2019 to 2021 was conducted at the Rice and Wheat Research Center of CSK Himachal Pradesh Krishi Vishvavidyalaya (HPKV) situated in Malan, within the North Western Himalayan region of Himachal Pradesh, India (as depicted in Fig. 1). This area is characterized by a sub-temperate humid climate, characterized by mild summers and harsh, freezing winters with sporadic snowfall. Notably, during the winter wheat crop growing season, the lowest mean temperature for the year 2019-20 was observed in January (as shown in Fig. 2), while in the subsequent year 2020-21, it occurred in December (as illustrated in Fig. 3). Additionally, during both of these research years, the month of May consistently recorded the highest mean weekly maximum temperature. Moreover, for both wheat growing seasons (2019-20 and 2020-21), May exhibited the highest average relative humidity, with values of 78.7% and 78.4% respectively, while the months of December saw the lowest relative humidity, registering values of 67.5% and 61.8% respectively.

**Fig. 4:**
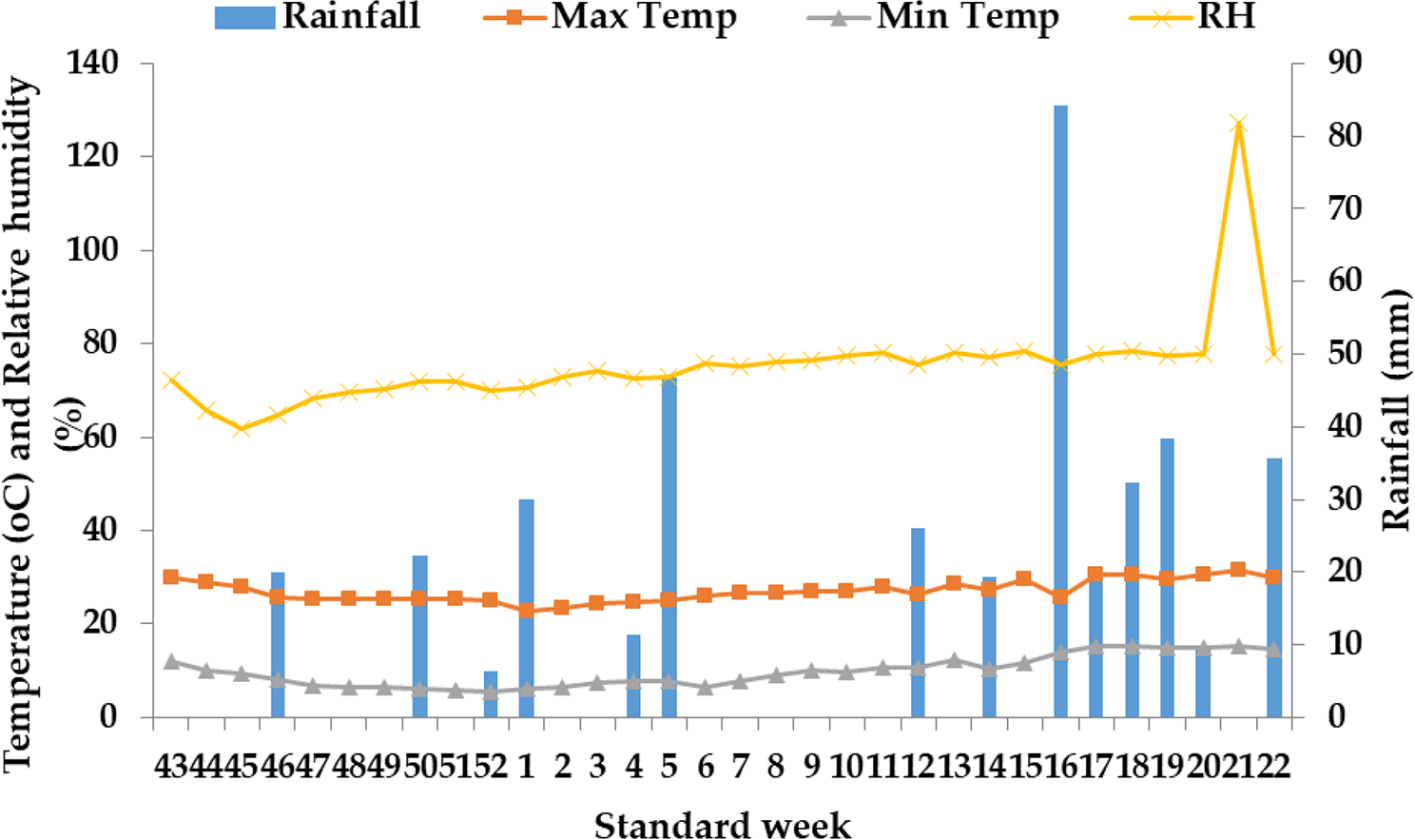
Mean weekly meteorological data at Malan during June 2020 to October 2020

**Fig. 5:**
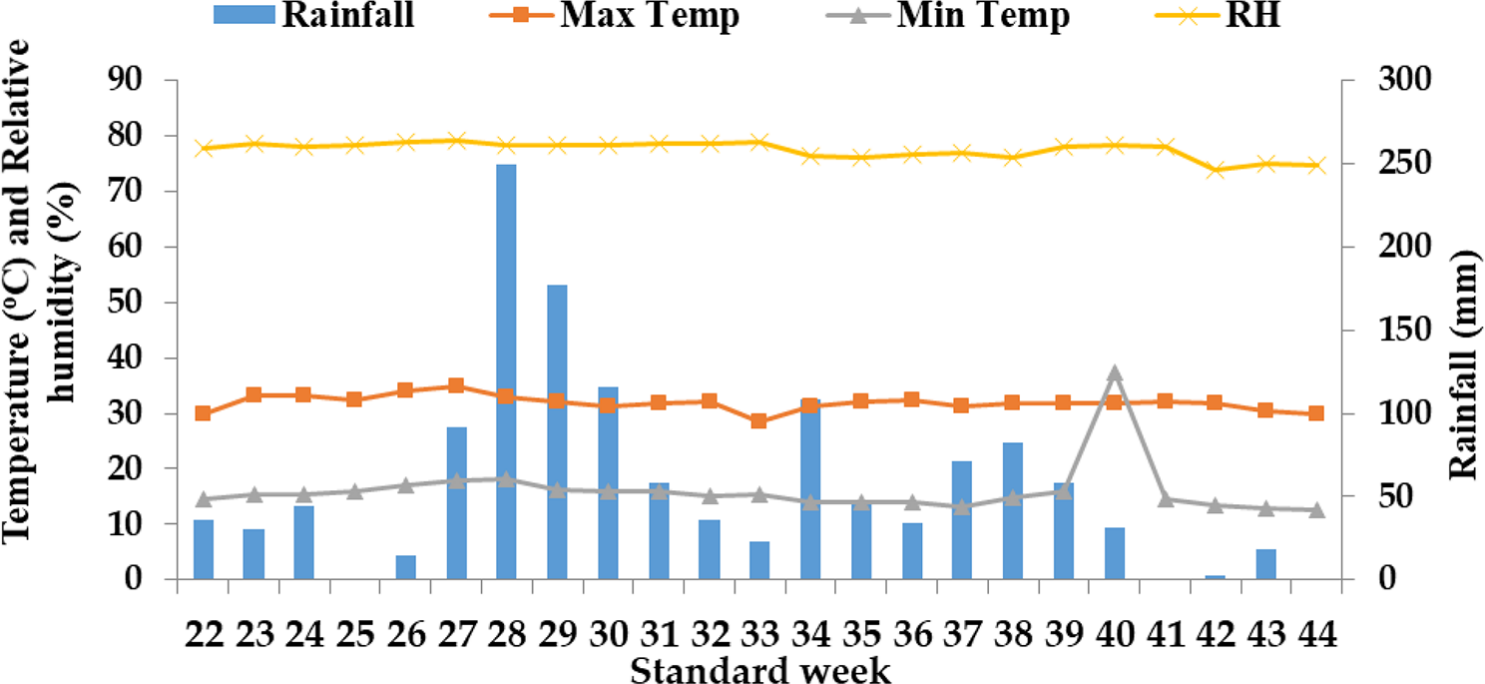
Mean weekly meteorological data at Malan during June 2021 to October 2021

Throughout the wheat growing seasons in both years, the crop received a consistent total rainfall of 120.4 mm in 2019-20 and 84.3 mm in 2020-21 (as indicated in Figs. 2 and 3). In contrast, during the rice crop growing season (which corresponds to the rainy season), the lowest mean temperature for the years 2020 and 2021 was recorded in the month of November (as shown in Figs. 4 and 5). During both rice crop seasons, the month of June consistently featured the highest mean weekly maximum temperature. Furthermore, during the wheat growing seasons of 2020 and 2021, the highest average relative humidity was observed in July, with values of 79.6% and 79.2% respectively, whereas the months of November showed the lowest relative humidity, with values of 65.6% and 74.7% respectively. It is worth noting that during the two wheat growing seasons in 2020 and 2021, the crops received a uniform total rainfall of 300.2 mm and 249.8 mm respectively (as indicated in Figs. 4 and 5). In terms of soil characteristics at the study site, the soil was found to be silty clay loam in texture, acidic in pH, and exhibited medium levels of available nitrogen, organic carbon, available phosphorus, and available potassium (as summarized in Table 1).

**Table 1:** Physico-chemical soil characteristics (0–15 cm depth) before beginning the experiment

Particular	Value	Analytical method employed
Soil	Silty clay loam	
pH	5.7	1:2.5 Soil water suspension method (Jackson, 1967) [25]
Organic carbon (OC)	10.2 g kg^− 1^	Rapid titration method (Walkley and Black, 1934) [26]
Available Nitrogen (N)	422.0 kg ha^− 1^	Alkaline permanganate method (Subbiah and Asija, 1956) [27]
Available Phosphorus (P)	17.8 kg ha^− 1^	Olsen’s method (Olsen et al., 1954) [28]
Available Potassium (K)	232.6 kg ha^− 1^	Neutral normal ammonium acetate extraction method (AOAC, 1970) [29]

### Experimental design and crop management

The field study was laid out in three repetitions in a split-plot pattern (width 2.3 m × length 2.4 m). The effect of two factors was analyzed:

- **Factor 1 (Main plot)** – Four cultivation methods: (1) reduced tillage (RT), (2) conventional tillage (CT), (3) natural farming (NF) and (4) zero tillage (ZT), (Table 2);
- **Factor 2 (sub plot) –** three genotypes each of rice: (1) *Him Palam Dhan* 1 (HPR 2656), (2) *Him Palam Lal Dhan* 1 (HPR 2795) and (3) *Sukara Dhan* 1 (HPR 1156) and wheat: (1) HPW 368 (2) HPW 349 and (3) HS 562.

The crop of wheat and rice was planted at 20 cm spacing on normal dates of sowing.

**Table:** 

Sowing date	Wheat	Rice
First season (2019-20)	08.12.2019	25.06.2020
Second season (2020-21)	29.11.2020	05.07.2021

Both wheat and rice were sown in the east-west row direction. There were 20 rows in each plot. Grain yield was determined from the central 16 lines comprising of net plot area. All experimental plots were treated with inorganic fertilizers at specific rates: 120 kg of nitrogen (N), 60 kg of phosphorus (P), and 40 kg of potassium (K) per hectare at the time of wheat sowing, and 60 kg of N, 30 kg of P, and 30 kg of K per hectare at the time of rice sowing. These fertilizers were applied in the form of urea, single super phosphate, and muriate of potash. Phosphorus and potassium were applied entirely during the planting stage, while nitrogen was applied in two equal portions at sowing and three weeks after sowing. The crop was cultivated following the recommended agricultural practices. Additionally, wheat and rice straw at a rate of 3 tons per hectare were used as mulch material and applied in the reduced tillage and natural farming treatments. In the case of natural farming treatment, all prescribed guidelines for such cultivation were strictly adhered to. The practices adopted in this treatment were given by natural farming expert Mr. Subhash Palekar [30]. The list of practices adopted in this treatment has been enumerated below:

1. Application of 500 kg ha^-1^ Ghanjeevamrit after grinding before sowing.
2. Dipping of rice and wheat seed in Beejamrit solution for 30 min before sowing.
3. Spray of Jeevamrit at one month after sowing by dissolving 25 L of Jeevamrit in 500 L water and using it in one ha area.
4. Second spray of Jeevamrit was done after 3 weeks of the first spray by dissolving 50 L of Jeevamrit in 500 L water and using it in one ha area.
5. The third spray of Jeevamrit was done after 3 weeks of the second spray by dissolving 50 L of Jeevamrit in 500 L water and using it in one ha area.
6. Spray of 25 L butter milk dissolved in 500 L water, 3 weeks after the last spray of Jeevamrit in one ha.

### Data recording

Data were collected following established protocols [31, 32]. Following the final harvest, the wheat and rice crops underwent sun-drying for a few days, after which their weights were recorded to determine the biological yield. Subsequently, the grain yield was documented. The straw yield was calculated by deducting the grain yield from the total biological yield. All recorded yields, including grain, straw, and biological yield, were converted into kilograms per hectare (kg ha^− 1^). For the assessment of nutrient concentrations, representative samples of both grain and non-grain above-ground portions were gathered at the time of harvest. These samples, pertaining to both grain and straw from both crop types, were then subjected to oven drying at 65 °C until a consistent dry weight was achieved. Following this, the samples were ground and utilized for the quantification of nitrogen, phosphorus, and potassium content in both the straw and grain (Table 3).

**Table 2:** Cultivation methods

Tillage	Cultivation measures
RT with residue (@ 3t ha^-1^)	Before planting the crop, only primary tillage was given, and the soil was amended with around 30% residue of the previous crop’s.
CT	Prior to sowing, optimal tilth in the field was achieved using both primary and secondary tillage.
NF	Sowing behind the country plough after two ploughings by power tiller
ZT	After the previous crop was harvested, a non-selective herbicide was applied to kill the weeds. A zero-till precision seed drill was then used to sow the subsequent crop.

**Table 3:** Analytical methods used for determination of chemical properties of the plant

Sr. No.	Chemical properties of plant samples	Method employed	Reference
1	Nitrogen	Micro-Kjeldahl	Jackson [34]
2	Phosphorus	Vanado-molybdo-phosphoric acid	Jackson [34]
3	Potassium	Flame photometer method	Richards [35]

### Soil sampling and analysis

Two sets of three replicate samples of surface (0–15 cm) soil layers were collected from all treatments before each crop was sown and after harvesting each crop with soil core sampler (with a core of 57 cm). The first set was used to measure bulk density of soil. The second set of soil samples were air dried, ground in a mortar and pestle, and sieved to pass through a 2 mm sieve. The soil was further analyzed for soil organic carbon through the wet digestion (rapid titration) method [26]. The available N content was determined by using the Alkaline permanganate method [27]. Available P content was determined using the Alkaline 0.5 NaHCO_3_ (pH 8.5) method [28] and available K concentration was measured using the Ammonium acetate extraction method [29–33].

### Statistical analysis

The data was examined using the OPSTAT statistical program and the analysis of variance (ANOVA; Table 4) approach as described by [36]. In order to assess and contrast the variations in standard error (SEm ±) and critical difference (CD) across different cultivation methods and genotype treatment averages, Fischer’s least significant test was utilized with a significance level of 5%.

**Table 4:** Analysis of variance for split-plot design

Source of variation	Degree of freedom
Replication	2
Cultivation methods (main plot)	3
Error (a)	6
Genotypes (subplot)	2
Cultivation methods x Genotypes (interaction)	6
Error (b)	16
Total	35

## Results and discussion

### Wheat crop

#### Cultivation methods and genotypes influence on yield attributes of wheat

Table 5 depict the results pertaining to the impact of cultivation methods and genotypes on the quantity of effective tillers per square metre and the number of grains per spike. Upon careful examination of the data, it became evident that cultivation methods and genotypes exerted a substantial impact on the quantity of effective tillers per square metre and the number of grains per spike. Conventional tillage exhibited notably elevated values of these parameters over the duration of the trial, which were comparable to those observed in reduced tillage. The natural farming treatment shown a notable decrease in the quantity of effective tillers per square metre and the number of grains per spike. The greater quantity of effective tillers per square metre and the larger number of grains per spike observed in conventional tillage can be attributed to the impact of cultivation on soil loosening, which enhances porosity and facilitates sufficient air exchange and root growth. Enhanced root development facilitates the plant’s ability to extract water and nutrients from a wider range of soil depths, leading to improved crop establishment and an increased tiller density per square metre. Similar finding indicating significantly higher values of different yield attributes of wheat under conventional tillage have also been reported by other workers [37, 38].

**Table 5:** Effect of cultivation methods on yield attributes of different wheat genotypes

Treatments	No. of effective tillers m^− 2^		No. of grains spike^− 1^		Grain weight (g) spike^− 1^		1000 grain weight (g)	
	2019-20	2020-21	2019-20	2020-21	2019-20	2020-21	2019-20	2020-21
**Main Plot Factor: Cultivation methods**								
Reduced tillage	310.6^ab^	294.6^ab^	53.65^ab^	49.64^ab^	2.29^ab^	2.18^ab^	41.84^ab^	42.35^ab^
Zero tillage	295.7^b^	278.7^b^	52.26^b^	47.75^b^	2.18^b^	2.08^b^	41.36^b^	41.96^b^
Conventional tillage	319.1^a^	307.5^a^	55.26^a^	50.90^a^	2.40^a^	2.23^a^	42.48^a^	42.76^a^
Natural farming	215.7^c^	227.5^c^	33.88^c^	34.46^c^	1.44^c^	1.37^c^	39.18^c^	38.8^c^
SEm ±	6.1	5.5	0.80	0.82	0.05	0.04	0.36	0.30
CD (*P* = 0.05)	21.1	18.9	2.78	2.84	0.18	0.13	1.24	1.04
**Sub Plot Factor: Genotypes**								
HPW 349	272.9^b^	268.9^b^	47.32^b^	44.18^b^	1.96^b^	1.86^b^	40.46^b^	41.08^b^
HPW 368	298.3^a^	288.1^a^	49.94^a^	46.94^a^	2.20^a^	2.06^a^	42.18^a^	42.09^a^
HS 562	284.6^ab^	274.2^ab^	49.03^ab^	45.94^ab^	2.07^ab^	1.94^ab^	41.00^b^	41.29^b^
SEm ±	4.7	5.2	0.60	0.70	0.05	0.05	0.25	0.22
CD (*P* = 0.05)	14.0	15.7	1.80	2.11	0.15	0.14	0.75	0.66

The figures presented are the average of three replications with four cultivation methods and three genotypes (combination 12). SEm ± or Standard error of the mean; CD-Critical difference; The mean that differ substantially among treatments are denoted by various letter in superscript

During the experimentation period, HS 562 demonstrated comparable performance to HPW 368 in terms of the number of effective tillers per square metre and the number of grains per spike. Notably, HS 562 consistently exhibited a significantly higher number of effective tillers per square metre and number of grains per spike compared to HPW 349, which displayed a significantly lower performance in these aspects (*P* < 0.05). The observed variations in the number of effective tillers per square metre and the number of grains per spike among genotypes can be related to the genetic factor of tillering potential exhibited by different genotypes.

Upon careful examination of the data presented in Table 5, it becomes evident that both cultivation methods and genotypes exerted a considerable influence on the weight of grains per spike. A notable decrease in grain weight per spike was observed in the natural farming treatment throughout both years of the study. Conversely, a significant increase in grain weight per spike was observed in the conventional tillage treatment, which was comparable to the reduced tillage treatment. The reduced tillage treatment, in turn, exhibited statistical similarity to the zero-tillage treatment. The decrease in grain weight per spike observed in the natural farming treatment can be attributed to the agricultural practices employed in this approach. These practices, although characteristic of natural farming, were unable to adequately fulfil the nutritional needs of the wheat crop. Consequently, the crop experienced suboptimal growth throughout the entire growing season, leading to the lowest grain weight per spike. The observed increase in grain weight per spike in the traditional tillage system may be attributed to enhanced crop growth resulting from improved soil physical and chemical characteristics, such as reduced bulk density, increased availability of macro and micronutrients, and improved aeration. Similar findings indicating significantly higher values of grain weight per spike of wheat under conventional tillage have also been reported by other workers [37]. Among the many genotypes that were evaluated, it was observed that HPW 368 exhibited a much greater grain weight per spike, which was comparable to HS 562 in both years of the study. Conversely, the wheat genotype HPW 349 shown a significantly lower grain weight per spike.

The influence of various treatments on 1000-grain weight is illustrated in Table 5. A review of the data presented in Table 5 reveals that conventional tillage resulted in a substantially greater 1000-grain weight than reduced tillage, with the latter treatment also being comparable to zero tillage during both study years. The natural farming treatment resulted in a significantly reduced 1000-grain weight. Conventional, reduced, and zero tillage treatments received the recommended dose of fertilizers, which ensured adequate and sustained nutrient supply throughout the crop cycle. This continuous availability of nutrients in these treatments led to greater photosynthate efficiency and dry matter accumulation, the remobilization of which to the grain was also facilitated by an adequate and regular supply of nitrogen and phosphorus, resulting in larger grains and a greater 1000-grain weight. The inability of natural farming practices to meet the nutrient needs of the wheat crop resulted in poor initial growth, reduced photosynthetic efficiency, and poor remobilization of photosynthates to the grain, which led to a significantly lower 1000-grain weight. Other workers have also reported significantly higher 1000-grain weight with conventional tillage [37, 38] as compared to other tillage methods.

The genotypes displayed considerable variance in terms of 1000-grain weight. During the course of the two-year experiment, it was seen that HPW 368 exhibited a much larger 1000-grain weight compared to other genotypes. In contrast, genotype HPW 349 shown a lower 1000-grain weight, but it was comparable to HS 562 in terms of this particular parameter.

#### Cultivation methods and genotypes influence on yield (kg ha^-1^) of wheat

**Fig. 6:**
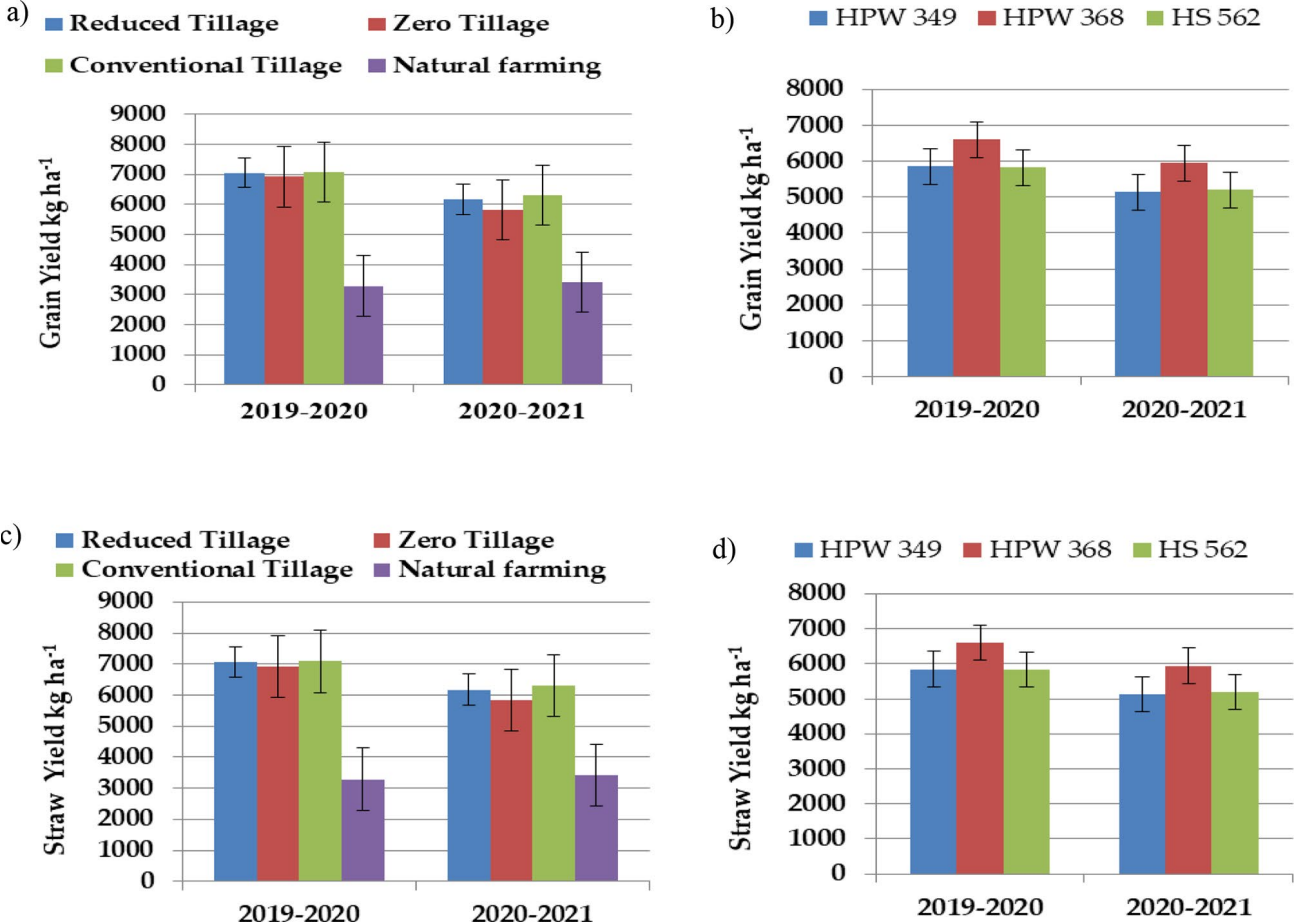
(**a**) and (**b**) Effect of cultivation methods on grain yield and (**c**) and (**d**) straw yield of different wheat genotypes. Error bars represent SE

Table 6; Fig. 6 depict the impact of various treatments on the grain production of wheat for the duration of the experimental period, encompassing both years. Throughout the duration of the study, it was shown that conventional tillage consistently yielded a much larger grain output compared to reduced tillage. This trend was observed consistently over the course of both experimental years. A notable decrease in wheat grain production was seen in the natural farming treatment. The observed increase in yield under conventional tillage can be attributed to the higher values of contributing characters or yield attributes in this particular treatment. During the process of conventional tillage, the soil undergoes a softening effect as a result of field preparation activities. Crop roots exhibit enhanced growth in soft soil in comparison to decreased and zero tillage practices. Enhanced root growth facilitates the extraction of a greater quantity of nutrients from the soil, thereby leading to improved growth and production outcomes within this particular treatment. The presence of reduced tillage residues on the soil surface can lead to a decrease in initial growth, primarily attributed to increased nitrogen immobilization, ultimately resulting in a reduced final yield. Similar finding indicating significantly higher values of grain yield of wheat under conventional tillage have also been reported by other workers [37–43].

**Table 6:** Effect of cultivation methods on yield (kg ha^− 1^) of different wheat genotypes

Treatments	Grain yield (kg ha^− 1^)		Straw yield (kg ha^− 1^)		Biological yield (kg ha^− 1^)		Harvest index (%)	
	2019-20	2020-21	2019-20	2020-21	2019-20	2020-21	2019-20	2020-21
**Main Plot Factor: Cultivation methods**								
Reduced tillage	4678^a^	4269^a^	7058^a^	6166^ab^	11,736^a^	10,435^ab^	39.87^a^	40.93^ab^
Zero tillage	4386^b^	3918^b^	6922^a^	5827^b^	11,308^a^	9745^b^	38.82^b^	40.27^b^
Conventional tillage	4825^a^	4503^a^	7084^a^	6301^a^	11,908^a^	10,804^a^	40.57^a^	41.73^a^
Natural farming	1979^c^	2164^c^	3287^b^	3411^c^	5265^b^	5574^c^	37.56^c^	38.83^c^
SEm ±	66	69	123	104	183	170	0.30	0.24
CD (*P* = 0.05)	228	240	427	359	634	591	1.04	0.83
**Sub Plot Factor: Genotypes**								
HPW 349	3743^b^	3501^c^	5848^b^	5131^b^	9591^b^	8633^b^	38.80^b^	40.33^b^
HPW 368	4174^a^	3925^a^	6592^a^	5944^a^	10,766^a^	9869^a^	38.45^b^	39.60^b^
HS 562	3984^a^	3713^b^	5822^b^	5203^b^	9806^b^	8917^b^	40.36^a^	41.40^a^
SEm ±	74	64	112	115	176	174	0.25	0.29
CD (*P* = 0.05)	222	192	336	344	527	523	0.76	0.87

The figures presented are the average of three replications with four cultivation methods and three genotypes (combination 12). SEm ± or Standard error of the mean; CD-Critical difference; The mean that differ substantially among treatments are denoted by various letter in superscript

Among the genotypes under investigation, it was found that the HPW 368 genotype exhibited a greater grain yield in wheat, whereas the HPW 349 genotype had a significantly lower yield in comparison. The HPW 368 genotype exhibited a higher yield as a result of an increased count of effective tillers per square metre, a greater number of grains per spike, and a higher test weight. These factors all contributed to the superior yield of this particular genotype [44, 45]. A decrease in the values of all these parameters in HPW 349 genotype led to a notable reduction in its yield.

An examination of the available data about the effects of cultivation techniques and genetic variations on the straw production of wheat indicated a noteworthy influence of both elements on this particular aspect. This observation is supported by the findings presented in Table 6; Fig. 6. The results indicated that conventional tillage resulted in a much greater straw production, followed by decreased tillage and zero tillage. The observed higher straw yield in conventional tillage practices may be attributed to factors such as enhanced nutrient availability and absorption, improved root growth, and increased photosynthetic activity, when compared to natural farming methods. Similar finding indicating significantly higher values of straw yield of wheat under conventional tillage have also been reported by other workers [43, 46]. Upon further examination of the data, it becomes evident that the natural farming treatment resulted in a significantly decreased straw yield of wheat. This reduction can be attributed to an inadequate supply of nutrients in the aforementioned treatment.

Among the genotypes that were examined, HPW 368 had the highest straw yield of wheat in both years, while the other two genotypes shown similar straw yields. The increased straw yield observed in HPW 368 can be attributed to its genotype’s enhanced tillering capacity, which facilitates more efficient utilization of available resources such as radiation and nutrients. Consequently, this genotype exhibits heightened photosynthetic activity, ultimately leading to a greater straw yield.

Table 6 displays the data pertaining to the influence of diverse cultivation methods on the biological yield of different wheat genotypes. Upon meticulous analysis of the data, it becomes evident that conventional tillage techniques continuously yielded much greater levels of biological productivity for wheat over the duration of the trial, spanning two years. Subsequently, the implementation of reduced tillage and zero tillage techniques ensued, whilst the natural farming approach continually shown noticeably diminished biological yields. The observed augmentation in biological production associated with traditional tillage can be ascribed to the enhanced aeration of the soil, which facilitates superior root development and fosters the availability and absorption of nutrients. Consequently, this results in enhanced initial growth and heightened photosynthetic activity.

In both years of the experiment, it was shown that HPW 368, among the various wheat genotypes investigated, exhibited significantly greater biological yields. Nonetheless, the study did not find any statistically significant disparities in biological yields between HS 562 and HPW 349, despite the fact that HPW 349 exhibited marginally lower biological yields in numerical terms. The increased biological yield of HPW 368 can be ascribed to its capacity for generating a greater quantity of tillers, hence resulting in an expanded leaf area. This expansion of leaf area then boosts photosynthetic activity, ultimately leading to elevated yields.

The data pertaining to the harvest index, as depicted in Table 6, exhibited notable disparities across various farming methods during both years of the study. The results repeatedly shown that conventional tillage led to a much higher harvest index, which was comparable to the harvest index achieved by decreased tillage practices. In contrast, natural farming treatment consistently yielded a much lower harvest index. The increased harvest index observed in conventional tillage practices can be attributed to the ample availability of nutrients during the flowering and maturity stages. This availability ensures the efficient translocation of photosynthates to the economically valuable portion of the plant, namely the grain, thereby leading to a higher harvest index. On the other hand, the natural farming treatment had a deficiency in nitrogen and phosphorus availability, particularly during the flowering stage. This deficiency resulted in inadequate translocation of photosynthates and subsequently led to a decreased harvest index. Among the genotypes that were examined, HS 562 exhibited a notably elevated harvest index, whilst the remaining two genotypes (HPW 368 and HPW 349) demonstrated comparable results. The harvest index is determined by the quantity of dry matter that is accumulated post-heading and subsequently remobilized to the grain. This index is primarily influenced by the genetic composition of the genotype.

#### Cultivation methods and genotypes influence on nutrient content of wheat

The data pertaining to the influence of various cultivation practice on the nitrogen content of several wheat genotypes is presented in Table 7. An analysis of the data indicated that the parameter in question was significantly affected by cultivation methods and genotypes. Throughout the two years of study, it was seen that the conventional tillage treatment resulted in notably greater levels of nitrogen content in both straw and grain compared to the decreased tillage condition. The results of the natural farming treatment indicate a notable decrease in nitrogen content observed in both the straw and grain samples during the two-year period. The increased nitrogen content seen in both straw and grain can be attributed to the larger application of nitrogen during the sowing period. This higher quantity of nitrogen likely led to its increased availability and subsequent uptake, particularly during the initial phases of crop growth. Moreover, the administration of increased levels of nitrogen and phosphorus during the sowing phase led to the development of a resilient and expansive root system. This root system likely facilitated the extraction of nitrogen from a broader range of soil depths, thus leading to elevated nitrogen content. In the context of natural farming, minimal amounts of nutrients were introduced into the soil, leading to an insufficient fulfilment of the crop’s nutritional needs. As a consequence, the growth and development of both the crop and its root system were adversely affected. The diminished presence of nitrogen in the soil, coupled with inadequate root development, may have contributed to a decrease in nitrogen levels observed in both the straw and grain. Similar finding indicating higher values of nitrogen content of wheat under conventional tillage have also been reported by other workers Saini et al. [43].

**Table 7:** Effect of cultivation methods on nitrogen, phosphorus and potassium content (%) of different wheat genotypes

Treatments	N content (%)				P content (%)				K content (%)			
	Grain		Straw		Grain		Straw		Grain		Straw	
	2019-20	2020-21	2019-20	2020-21	2019-20	2020-21	2019-20	2020-21	2019-20	2020-21	2019-20	2020-21
**Main Plot Factor: Cultivation methods**												
Reduced tillage	1.49	1.47	0.46	0.44	0.262	0.248	0.109	0.104	0.320	0.340	1.260	1.280
Zero tillage	1.46	1.45	0.45	0.43	0.258	0.243	0.107	0.102	0.320	0.340	1.240	1.270
Conventional tillage	1.53	1.51	0.46	0.44	0.268	0.254	0.112	0.107	0.330	0.350	1.300	1.330
Natural farming	1.42	1.38	0.43	0.41	0.246	0.227	0.104	0.098	0.300	0.320	1.210	1.230
SEm ±	0.02	0.02	0.006	0.005	0.003	0.004	0.001	0.002	0.004	0.005	0.02	0.02
CD (*P* = 0.05)	0.07	0.06	0.02	0.02	0.011	0.015	0.005	0.006	0.01	0.02	0.06	0.08
**Sub Plot Factor: Genotypes**												
HPW 349	1.46	1.43	0.44	0.43	0.255	0.237	0.107	0.104	0.320	0.340	1.240	1.300
HPW 368	1.49	1.45	0.45	0.43	0.259	0.244	0.107	0.101	0.310	0.330	1.250	1.260
HS 562	1.47	1.48	0.46	0.44	0.261	0.248	0.110	0.103	0.320	0.340	1.270	1.270
SEm ±	0.02	0.03	0.005	0.006	0.003	0.003	0.001	0.001	0.003	0.004	0.02	0.03
CD (*P* = 0.05)	NS	NS	NS	NS	NS	0.009	NS	NS	NS	NS	NS	NS

The results of the experiment indicate that genotypes did not exert a statistically significant effect on the nitrogen content in both straw and grain during the duration of the study.

Table 7 presents the data pertaining to the phosphorus concentration in straw and grain, with consideration given to the influence of cultivation methods and genotypes. The results indicate a notable degree of variance resulting from the aforementioned factors. Additionally, it was shown that the phosphorus concentration in grain exceeded that in straw, potentially indicating the transfer of phosphorus from the shoot to the grain during the grain filling stage [51]. A notable decrease in phosphorus concentration was seen in both straw and grain under the natural farming treatment in both years. Conversely, a considerable increase in phosphorus concentration was observed in straw and grain under the conventional tillage treatment, which was comparable to the reduced tillage treatment in both years. The observed increase in phosphorus content in both straw and grain under various tillage treatments compared to the natural farming treatment may be attributed to the use of the appropriate dosage of nutrients in the tillage treatments, whilst no chemical fertilizer was utilized in the natural farming treatment. The implementation of the recommended dosage of phosphorus in tillage treatments led to an augmentation in the accessibility of this nutrient in the soil. This, in conjunction with the heightened availability of nitrogen, resulted in the development of a more robust and expansive root system. Consequently, the roots were able to explore a broader range of soil, leading to a higher concentration of phosphorus in both the straw and grain. Similar findings indicating higher values of phosphorus concentration of wheat under conventional tillage have also been reported by other workers [43] and [46].

The genotypes did not demonstrate a significant impact on the phosphorus content in straw and grain throughout the duration of the experiment, with the exception of the grain content in the 2020-21 period. The genotype HS 562 exhibited a notably greater phosphorus content in grain, which was shown to be statistically equivalent to the phosphorus level observed in genotype HPW 368. In turn, the phosphorus content in genotype HPW 368 was statistically equivalent to that of genotype HPW 349, which displayed a much lower phosphorus content in grain.

Table 7 presents the potassium levels in wheat straw and grain, with consideration given to the influence of production methods and genotypes. Upon reviewing the data, it is evident that the potassium concentration was greatly influenced by the growing methods employed. The potassium concentration in straw and grain was found to be significantly greater in the conventional tillage system, which was comparable to the levels observed in reduced tillage and zero tillage systems. A notable decrease in the potassium concentration was seen in the straw and grain samples subjected to the natural farming treatment during both years of the experimental study. The elevated potassium levels seen in the tillage plots can be attributed to the application of a larger quantity of potassium in these treatments. This increased application may have enhanced the availability of potassium in the soil, subsequently leading to higher potassium content in both the straw and grain. Moreover, the application of fertilizer may have resulted in the development of deep and extensive root systems, which could have led to the extraction of potassium from a broader range of soil, thus resulting in higher potassium concentration. Similar findings indicating higher values of potassium concentration of wheat under conventional tillage have also been reported by other workers [43]. The potassium level observed in the straw and grain of several wheat genotypes remained unaffected in a statistically significant manner during both years of study.

### Rice crop

#### Cultivation methods and genotypes influence on yield attributes of rice

The data pertaining to the influence of cultivation methods and rice genotypes on the number of panicles per square metre (m^− 2^) is presented in Table 8. The findings presented in this study illustrate the substantial impact of both variables on the specific parameter under investigation. Upon conducting a more thorough analysis of the data, it becomes apparent that the natural farming treatment consistently displayed a significantly reduced quantity of panicles per square metre and grains per panicle during both years of the study. The decline in rice crop yield can be ascribed to the inadequate provision of crucial nutrients, including nitrogen and phosphorus. Among the several cultivation methods that were examined, it was observed that traditional tillage exhibited a significantly greater quantity of panicles per square metre and grains per panicle. These findings were found to be very comparable to the outcomes produced through the implementation of reduced tillage. The observed higher panicle density and grain yield per panicle in conventional tillage practices can be linked to the effects of soil loosening, improved soil porosity, and the facilitation of optimal air exchange and root growth. Enhanced root development facilitates increased nutrient and water uptake by rice plants from a wider range of soil depths, leading to improved crop establishment and higher values in terms of panicle density per square metre and grain yield per panicle. In relation to the rice genotypes being studied, it was seen that the genotype “Him Palam Lal Dhan 1” demonstrated a statistically significant increase in the number of panicles per square metre and grains per panicle compared to the remaining genotypes. The genotypes “Sukara Dhan 1” and “Him Palam Dhan 1” also displayed similar trends, albeit to a little lesser extent. The observed variations among the genotypes with respect to this metric can be ascribed to their genetic constitution. Similar differences among different rice genotypes were also reported by number of workers [37, 43, 48, 49].

**Table 8:** Effect of cultivation methods on yield attributes of different rice genotypes

Treatments	No. of panicles m^− 2^		No. of grains panicle^− 1^		Grain weight panicle^− 1^		1000 grain weight (g)	
	2020	2021	2020	2021	2020	2021	2020	2021
**Main Plot Factor: Cultivation methods**								
Reduced tillage	269.3	284.0	76.7	78.9	1.92	1.89	22.66	23.32
Zero tillage	255.9	268.7	73.4	73.7	1.80	1.82	22.40	23.08
Conventional tillage	281.7	295.3	78.9	81.9	1.99	1.98	22.80	23.56
Natural farming	226.8	244.8	61.2	66.3	1.48	1.58	20.72	21.92
SEm ±	5.6	6.2	1.4	1.8	0.04	0.04	0.16	0.20
CD (*P* = 0.05)	19.3	21.5	4.7	6.3	0.13	0.14	0.56	0.69
**Sub Plot Factor: Genotypes**								
*Sukara Dhan* 1 (HPR 1156)	257.3	272.3	72.7	75.9	1.79	1.84	21.74	22.95
*Him Palam Dhan* 1 (HPR 2656)	249.1	264.4	70.0	71.3	1.74	1.72	21.96	22.60
*Him Palam Lal Dhan* 1 (HPR 2795)	268.9	282.9	74.9	78.4	1.86	1.89	22.74	23.36
SEm ±	4.3	4.0	1.3	1.5	0.03	0.03	0.14	0.17
CD (*P* = 0.05)	12.9	12.0	3.8	4.6	0.09	0.10	0.42	0.50

Upon analyzing the data provided in Table 8, it was observed that both the cultivation methods and genotypes exerted a substantial influence on the weight of grains per panicle. The experimental findings consistently indicated that the implementation of natural farming practices resulted in a considerable decrease in grain weight per panicle across both years of the study. In contrast, the conventional tillage method, in conjunction with reduced tillage, consistently yielded considerably greater grain weight per panicle. After doing a more thorough examination of the data, it became apparent that the observed disparities between reduced tillage and zero tillage methods did not achieve statistical significance in relation to the grain weight per panicle during both experimental years. The absence of statistical significance indicates that both tillage methods exhibited comparable impacts on grain weight per panicle over the duration of the trial. As previously mentioned, the methodologies utilized in natural farming proved inadequate in meeting the nutritional demands of the rice crop, leading to suboptimal crop development for the entirety of the cultivation period. The deficient growth finally resulted in the lowest documented grain weight per panicle. The increased grain weight per panicle observed in conventional tillage practices can be linked to the enhancement of soil physical and chemical features, such as a decrease in bulk density and an increase in the accessibility of macro and micronutrients. The observed enhancements can be ascribed to the accelerated breakdown of agricultural residue during the course of the crop cycle. Other workers have also reported significantly higher grain weight per panicle with conventional tillage [37, 43, 47] as compared to other tillage methods. Statistically significant variations were noted across several genotypes in terms of grain weight per panicle. Him Palam Lal Dhan 1 exhibited a significantly larger grain weight per panicle (1.86 and 1.89 g) compared to the other two genotypes, Sukara Dhan 1 (1.79 and 1.84 g) and Him Palam Dhan 1(1.74 and 1.72 g), which showed similar grain weights.

The data depicted in Table 8 provides an illustration of the influence that various cultivation methods and genotypes have on the 1000-grain weight. After doing an analysis of the data, it is apparent that conventional tillage resulted in a noticeably greater 1000-grain weight, a finding that aligns with the outcomes seen in both reduced tillage and zero tillage conditions. In contrast, the natural farming treatment had a notably reduced value for this measure.

The observed higher 1000-grain weight in all three tillage treatments, in contrast to natural farming, can be ascribed to the use of the recommended dosage of fertilizers in these treatments. The utilization of this particular application resulted in a continuous and abundant provision of essential nutrients to the crop during its whole growth and development process, leading to an improvement in the efficiency of photosynthesis and an increase in the production of dry matter. Moreover, the sufficient availability of nitrogen and phosphorus is believed to have played a role in facilitating an enhanced and effective translocation of photosynthates to the grains, resulting in the development of larger and heavier grains, and thus, an elevated 1000-grain weight. Other workers have also reported significantly higher 1000-grain weight with conventional tillage [43, 48, 49] as compared to other tillage methods.

The genotypes shown notable diversity in terms of 1000-grain weight. The genotype Him Palam Lal Dhan 1 exhibited the greatest recorded 1000-grain weight, whereas the genotypes Him Palam Dhan 1 and Sukara Dhan 1 demonstrated similar results. The primary determinant of the 1000-grain weight of a genotype is mostly influenced by the genetic composition of the genotype, which accounts for the observed variations.

#### Cultivation methods and genotypes influence on yield (kg ha^-1^) of rice

**Fig. 7:**
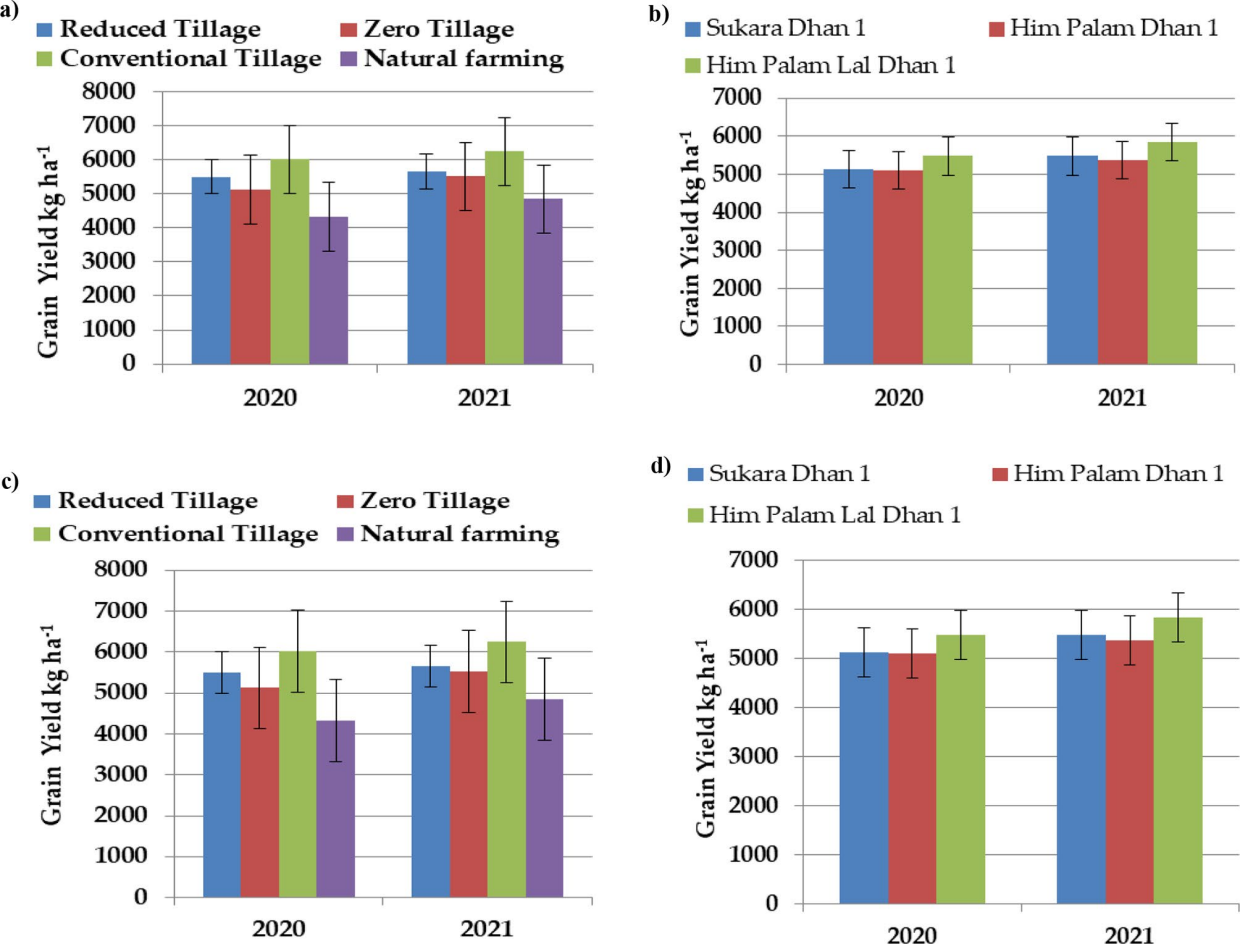
(**a**) and (**b**) Effect of cultivation methods on grain yield and (**c**) and (**d**) straw yield of different rice genotypes. Error bars represent SE

The data shown in Table 9; Fig. 7 illustrates the influence of different cultivation methods and genotypes on the production of rice grains. The results of the two-year experimental study clearly demonstrate that both cultivation methods and genotypes exerted a substantial impact on rice yield. The findings consistently indicated that conventional tillage practices consistently yielded the maximum grain production, followed by reduced tillage and zero tillage methods. Conversely, natural farming consistently yielded much lower rice yields.

**Table 9:** Effect of cultivation methods on yield (kg ha^− 1^) of different rice genotypes

Treatments	Grain yield		Straw yield		Biological yield		Harvest index	
	2020	2021	2020	2021	2020	2021	2020	2021
**Main Plot Factor: Cultivation methods**								
Reduced tillage	3060	3213	5498	5656	8559	8869	35.74	36.18
Zero tillage	2778	3051	5122	5517	7899	8568	35.14	35.58
Conventional tillage	3421	3587	6013	6247	9434	9834	36.25	36.45
Natural farming	2281	2602	4326	4847	6607	7450	34.50	34.91
SEm ±	69	66	121	114	189	176	0.18	0.20
CD (*P* = 0.05)	238	229	419	397	655	608	0.62	0.69
**Sub Plot Factor: Genotypes**								
*Sukara Dhan* 1 (HPR 1156)	2880	3126	5133	5477	8013	8603	35.86	36.27
*Him Palam Dhan* 1 (HPR 2656)	2705	2865	5104	5376	7809	8242	34.55	34.73
*Him Palam Lal Dhan* 1 (HPR 2795)	3070	3348	5482	5847	8553	9195	35.82	36.25
SEm ±	67	60	108	105	171	161	0.07	0.07
CD (*P* = 0.05)	200	182	325	314	512	484	0.23	0.22

Conventional tillage encompasses the combined practices of primary and secondary tillage, which aim to establish favourable soil conditions inside the root zone. The promotion of appropriate soil tilth facilitates improved crop germination and the establishment of a robust root system, enabling roots to effectively reach a broader and deeper expanse of soil for enhanced nutrient uptake, particularly in relation to nitrogen and phosphorus. The augmentation of nutrient absorption results in elevated tillering, enhanced seed set, and eventually greater values in relation to qualities associated with yield and grain production. In addition, the heightened accessibility of nitrogen contributes to the prolonged maintenance of leaf greenness, leading to enhanced photosynthetic efficiency and subsequently, greater crop yields. Similar finding indicating significantly higher values of grain yield of rice under conventional tillage have also been reported by other workers [20, 38, 42, 43, 50].

In relation to the various rice genotypes being examined, it was seen that Him Palam Lal Dhan 1 demonstrated a significantly greater grain yield in comparison to Him Palam Dhan 1, which revealed a notably diminished yield. The enhanced productivity of Him Palam Lal Dhan 1 can be ascribed to its larger panicle density per unit area, increased grain count per panicle, and elevated test weight within this specific genetic variant. The combined influence of these parameters led to the increased yield observed in Him Palam Lal Dhan 1, while the lower values of these characteristics in Him Palam Dhan 1 resulted in its diminished yield performance.

The findings depicted in Table 9; Fig. 7 illustrate that the straw yield of rice is significantly affected by both cultivation methods and genotypes during the two-year experimental period. The results consistently showed that conventional tillage resulted in the highest straw output, followed closely by decreased tillage and zero tillage. Interestingly, the latter two approaches demonstrated similar outcomes in both experimental years. The increased straw production observed in conventional tillage can be attributable to several factors, including better root growth, improved nutrient availability and uptake, and elevated photosynthetic activity, in comparison to natural farming practices. Similar finding indicating significantly higher values of straw yield of rice under conventional tillage have also been reported by other workers [21, 49, 50]. Upon further analysis of the data, it becomes evident that the implementation of the natural farming strategy led to a notable decrease in straw output. This decline may be primarily attributed to insufficient nutrient provision within this specific treatment. Regarding the genotypes being examined, it was observed that Him Palam Lal Dhan 1 consistently demonstrated a much greater straw yield of rice, whereas Him Palam Dhan 1 consistently exhibited a significantly lower straw yield across both years of the test.

The biological yield of rice is presented in Table 9, showcasing the influence of cultivation methods and genotypes. The findings of the data analysis indicate that the utilization of conventional tillage practices had a notable positive impact on the biological production of rice, whereas the adoption of natural farming methods resulted in the lowest output. The factors contributing to the enhanced effectiveness of conventional tillage have been previously examined.

In the investigation of different genotypes, it was continuously seen that Him Palam Lal Dhan 1 demonstrated the highest biological yield over both years of study (2020 and 2021). Following closely behind were the genotypes Sukara Dhan 1 and Him Palam Dhan 1, which exhibited similar yields. Similar finding indicating significantly higher values of biological yield of rice under conventional tillage have also been reported by other workers [21, 38, 49].

The data on the harvest index is likewise displayed in Table 9, indicating that it was considerably impacted by both cultivation methods and genotypes over the two years of testing. The harvest index values were seen to be significantly greater in the conventional tillage and reduced tillage treatments, but the natural farming treatment exhibited a much lower index. The harvest index, which quantifies the proportion of economic yield in relation to the overall biomass yield, is mostly determined by genetic and environmental factors rather than agronomic practices. As a result, there were only minor fluctuations observed in the harvest index. Nevertheless, variations in cultivation techniques were ascribed to the presence of essential nutrients, specifically nitrogen and phosphorus, and their contributions to the production of dry matter and its efficient transportation to the grains. The utilization of conventional tillage practices, characterized by a greater abundance of nutrients, has been found to promote enhanced crop yield and improved transportation of photosynthates, ultimately leading to a higher harvest index.

In contrast, the harvest index of several rice genotypes exhibited variation, with Sukara Dhan displaying a much higher harvest index compared to Him Palam Lal Dhan 1, while the latter was comparable. Conversely, Him Palam Dhan exhibited a significantly lower harvest index. The variations in harvest index observed among different genotypes can be ascribed to the genetic composition of these kinds. Other workers have also reported significantly higher harvest index with conventional tillage [21, 43, 49] as compared to other tillage methods.

#### Cultivation methods and genotypes influence on nutrient content of rice

Table 10 presents the data about the impact of cultivation methods on the nitrogen content in straw and grain of various rice genotypes. The findings indicate a significant influence of both cultivation methods and genotypes. The nitrogen content in grain was found to be notably greater during both years under conventional tillage, comparable to that of reduced tillage and zero tillage. The application of natural farming techniques resulted in a notable reduction in the nitrogen content seen in the grain samples throughout the course of both years. In relation to the nitrogen content found in straw, it was seen that conventional tillage demonstrated comparable results to reduced tillage and zero tillage practices, with all three methods exhibiting much higher nitrogen content in straw. Conversely, the natural farming treatment exhibited significantly lower nitrogen content in straw. The increased nitrogen content observed in both straw and grain under various tillage treatments can be attributed to the application of acceptable doses of fertilizers during sowing. This application likely led to higher nitrogen availability, facilitating its uptake, especially during the initial stages of crop growth. Moreover, the administration of increased levels of nitrogen and phosphorus during the sowing phase yielded a resilient and expansive root system, perhaps facilitating the extraction of nitrogen from a broader soil profile. Consequently, this phenomenon led to an elevated nitrogen content. In the context of natural farming, little nutrients were introduced into the soil, leading to an insufficient supply for the crop’s needs. Consequently, this inadequacy resulted in suboptimal growth and a compromised root structure. The diminished presence of nitrogen in the soil, coupled with inadequate root development, may have contributed to a decrease in nitrogen levels observed in both the grain and straw. Similar finding indicating higher values of nutrient content of rice under conventional tillage have also been reported by Saini et al. [43].

**Table 10:** Effect of cultivation methods on nitrogen, phosphorus and potassium content (%) of different rice genotypes

Treatments	N content (%)				P content (%)				K content (%)			
	Grain		Straw		Grain		Straw		Grain		Straw	
	2019-20	2020-21	2019-20	2020-21	2019-20	2020-21	2019-20	2020-21	2019-20	2020-21	2019-20	2020-21
**Main Plot Factor: Cultivation methods**												
Reduced tillage	1.37	1.41	0.47	0.48	0.277	0.286	0.118	0.123	0.216	0.222	1.197	1.189
Zero tillage	1.35	1.40	0.46	0.47	0.267	0.279	0.114	0.118	0.209	0.215	1.186	1.180
Conventional tillage	1.38	1.42	0.47	0.49	0.285	0.297	0.125	0.133	0.219	0.228	1.219	1.205
Natural farming	1.30	1.35	0.43	0.44	0.259	0.256	0.105	0.111	0.204	0.207	1.140	1.141
SEm ±	0.009	0.010	0.007	0.006	0.005	0.006	0.002	0.002	0.003	0.002	0.007	0.006
CD (*P* = 0.05)	0.03	0.03	0.02	0.02	0.017	0.020	0.005	0.006	0.011	0.008	0.025	0.021
**Sub Plot Factor: Genotypes**												
*Sukara Dhan* 1 (HPR 1156)	1.34	1.39	0.47	0.48	0.271	0.276	0.116	0.122	0.213	0.219	1.192	1.177
*Him Palam Dhan* 1 (HPR 2656)	1.34	1.38	0.46	0.47	0.268	0.271	0.111	0.117	0.208	0.216	1.190	1.172
*Him Palam Lal Dhan* 1 (HPR 2795)	1.36	1.41	0.45	0.46	0.278	0.291	0.120	0.126	0.216	0.219	1.175	1.187
SEm ±	0.008	0.009	0.004	0.005	0.003	0.002	0.002	0.002	0.002	0.002	0.005	0.004
CD (*P* = 0.05)	NS	NS	0.01	NS	0.008	0.006	0.006	0.005	0.005	NS	0.015	0.013

The nitrogen content in grain was observed to be consistent among the genotypes studied across both years of research. However, a notable disparity in nitrogen content inside the straw was observed solely during the year 2020. The nitrogen concentration in straw for the year 2020 was found to be higher in the Sukara Dhan 1 genotype, which was comparable to the Him Palam Dhan 1 genotype, and in turn, was comparable to the Him Palam Lal Dhan 1 genotype. The increased nitrogen content observed in the Genotypes can be attributed to their more developed root system, which effectively collected nutrients, particularly nitrogen, from a greater soil profile. The potential influence of genetic composition on the observed difference in nitrogen concentration among various varieties must be overlooked.

Table 10 presents the data pertaining to the phosphorus content in straw and grain, with consideration given to the impact of cultivation methods and genotypes. The results indicate notable variations attributed to both factors. Additionally, it was observed that the phosphorus concentration in grain was greater than that in straw, potentially indicating the movement of phosphorus from the shoot to the grain during the grain filling stage [51]. A notable decrease in phosphorus content was observed in both straw and grain samples under the natural farming treatment throughout the duration of the study. The underlying cause for this phenomenon has been previously addressed. The phosphorus content in straw and grain was found to be notably higher in the conventional tillage system, comparable to the reduced tillage system in 2020, and comparable to both the reduced tillage and zero tillage systems in 2021. Higher phosphorus content in grain and straw was attributable to higher fertilizer application in tillage plots, which may have led in to the enhanced availability of phosphorus in the soil, particularly during the first phases of crop growth. Also, the greater availability of phosphorus in the soil could have resulted in the establishment of a deep and extended root system and improved early growth which al-lowed to roots to explore the larger soil profile for nutrients resulting in higher nutrient content and uptake. Archana et al. [52] have also documented an increase in phosphorus content and uptake by the rice crop when higher levels of phosphorus were applied.

The genotype Him Palam Lal Dhan 1 exhibited the highest phosphorus level in both straw and grain, while the genotype Him Palam Dhan 1 had the lowest phosphorus content in both straw and grain. The observed variations in phosphorus content in straw and grain among different genotypes may be attributed to internal or external mechanisms that facilitate enhanced soil phosphorus extraction and grain production [53].

Table 10 provides information on the potassium level in rice straw and grain, which is regulated by cultivation methods and genotypes. Upon examination of the data, it was observed that the cultivation methods exerted a noteworthy impact on the potassium levels found in both the straw and grain. The potassium content in straw and grain was found to be notably greater in the conventional tillage treatment compared to both zero tillage and reduced tillage during the initial year (2020). Additionally, during the second year (2021), the potassium content in the conventional tillage treatment was at par with reduced tillage only. The natural farming treatment exhibited the most notable decrease in potassium levels in both grain and straw. The elevated levels of potassium seen in the tillage plots can be attributed to the use of the recommended dosage of fertilizers in these treatments. This application potentially enhanced the availability of potassium in the soil, leading to increased potassium content in both the straw and grain. Furthermore, the use of traditional tillage practices resulted in the development of deep and extended root systems. This may be attributed to the improved physical condition and aeration of the soil. As a consequence, the roots were able to collect potassium from a broader range of soil layers, ultimately leading to an increased potassium content. The crop cultivated using natural farming methods exhibited suboptimal initial growth, potentially attributable to insufficient nutrient availability. Consequently, the compromised growth, particularly in the root system, hindered the uptake of nutrients from the entire soil profile, resulting in a deficient potassium content in the grains. Similar finding indicating significantly higher values of potassium content in rice under conventional tillage have also been reported by other workers [43].

Genotypes also behaved differently with respect to potassium content in grain and straw. Significantly higher potassium content in grain during 2020 was observed in Him Palam Lal Dhan 1 though it was at par with Sukara 6Dhan 1 which in turn was also at par with Him Palam Dhan 1. Potassium content in grain during 2021 was not affected significantly. Regarding potassium content in straw during 2020 significantly higher potassium content was recorded in Sukara Dhan 1 while significantly lowest potassium content was recorded in Him Palam Lal Dhan 1. In 2021 the trend was reversed with significantly higher potassium content recorded in Him Palam Lal Dhan 1 while significantly lower potassium content was recorded in Him Palam Dhan 1.

### Soil chemical properties

The examination of the data reported in Table 11 indicated that the cultivation methods had a substantial impact on the available soil nitrogen levels following crop harvest, with the exception of the winter season of 2019-20. However, the genotypes did not exhibit a significant influence on this particular parameter for either of the two years. The maximum availability of nitrogen (N) was observed with reduced tillage following harvest. This can be attributed to several factors, including the decomposition and mineralization of integrated crop residues, improved soil aeration, and increased microbial activity. These factors likely contribute to the overall increase in nitrogen availability. The availability of soil nutrients and the uptake of nutrients by crops are significant considerations in the process of residue mineralization. Zhu et al. [54] also reported comparable findings. The lowest nitrogen availability was seen in the context of natural farming, which can be attributed to the degradation of soil structure in natural farming and conventional tillage practices. It is possible that the greater rates of mineralization and/or leaching contribute to the decline in nitrogen availability in tilled plots. Singh et al. [55] obtained comparable findings in their investigation, which indicated a reduced availability of nitrogen (N) associated with conventional ploughing practices. This decrease in N availability might be related to the process of topsoil inversion during ploughing, which brings less fertile subsoil to the surface, together with the occurrence of leaching. There was a lack of statistically significant impact observed on the nitrogen availability for the genotypes examined in both years of the study. The study determined that the impact of cultivation methods and genotypes on the availability of nitrogen (N) was not statistically significant.

**Table 11:** Effect of cultivation methods and genotypes on available nitrogen, phosphorus, potassium and organic carbon

Treatments	Available N (kg ha^− 1^)				Available P (kg ha^− 1^)				Available K (kg ha^− 1^)				Soil organic carbon (g kg^− 1^)			
	After winter 2019-20	After Rainy 2020	After winter 2020-21	After Rainy 2021	After winter 2019-20	After Rainy 2020	After winter 2020-21	After Rainy 2021	After winter 2019-20	After Rainy 2020	After winter 2020-21	After Rainy 2021	After winter 2019-20	After Rainy 2020	After winter 2020-21	After Rainy 2021
**Main Plot Factor: Cultivation methods**																
Reduced tillage	424.3	426.8	429.8	433.7	19.0	19.2	19.8	20.1	234.6	231.7	229.5	235.3	8.86	8.91	8.97	9.03
Zero tillage	421.5	422.2	420.7	418.2	18.6	18.2	19.0	19.4	228.6	225.9	223.4	220.2	8.83	8.89	8.94	8.99
Conventional tillage	418.7	415.3	413.7	408.5	17.9	7.3	17.6	17.5	227.5	223.8	219.8	216.4	8.77	8.72	8.70	8.67
Natural farming	415.1	408.2	400.4	396.2	17.0	15.9	15.6	15.4	226.8	219.5	215.7	210.8	8.84	8.86	8.91	8.94
SEm ±	2.8	3.0	3.0	3.1	0.3	0.4	0.4	0.4	2.0	2.2	2.2	2.3	0.05	0.06	0.06	0.07
CD (*P* = 0.05)	NS	10.3	10.4	10.7	1.2	1.3	1.5	1.5	6.8	7.5	7.7	8.0	NS	0.20	0.22	0.25
**Sub Plot Factor: Genotypes**																
V_1_	422.0	417.5	417.6	413.9	18.4	17.2	17.7	17.8	229.6	225.1	222.8	219.4	8.81	8.82	8.84	8.87
V_2_	417.5	418.9	415.1	415.6	17.9	18.0	17.9	18.3	228.1	224.5	220.1	221.0	8.84	8.86	8.91	8.94
V_3_	420.2	418.3	415.8	412.9	18.1	17.8	18.4	18.2	230.4	226.1	223.4	221.6	8.83	8.86	8.89	8.91
SEm ±	2.2	2.1	2.0	2.2	0.3	0.4	0.3	0.3	1.4	1.3	1.4	1.3	0.02	0.02	0.03	0.03
CD (*P* = 0.05)	NS	NS	NS	NS	NS	NS	NS	NS	NS	NS	NS	NS	NS	NS	NS	NS

Initial value, N: 422.0 Initial value, P: 17.8 Initial value, K: 232.6 Initial value, SOC: 8.80

V1: HPW 349 (Wheat) and *Sukara Dhan* 1 (Rice)

V2: HPW 368 (Wheat) and *Him Palam Dhan*1 (Rice)

V3: HS 562 (Wheat) and *Him Palam Lal Dhan*1 (Rice)

The data pertaining to the impact of different treatments on the availability of phosphorus in soil revealed notable variations as a result of tillage practices. However, the influence of wheat and rice types did not yield statistically significant results, as indicated in Table 11. During all the cropping seasons, decreased tillage and zero tillage exhibited comparable levels of accessible phosphorus, with significantly higher amounts seen in both tillage practices. The increased availability of phosphorus in this particular treatment may be attributed to the accumulation of inorganic phosphorus resulting from the integration and decomposition of organic wastes in the reduced tillage approach. The practice of natural farming resulted in the observed lowest availability of phosphorus (P). This can be attributed to the absence of chemical fertilizers and the limited application of P to the soil. In contrast, soils subjected to minimum tillage exhibited higher levels of extractable P compared to both natural farming and conventional tilled soil. This can be attributed to the reduced mixing of fertilizer P with the soil, leading to a lower degree of phosphorus fixation. The increased presence of phosphorus (P) in zero tillage systems compared to conventional tillage and natural farming can be attributed to the process of organic element mineralization occurring at the soil surface. This mineralization process serves as a significant contributor to the availability of accessible nutrients. The findings of Bhatt [56], yielded similar outcomes. No substantial impact on available phosphorus was seen across the several genotypes evaluated in both years of the experimental investigation.

The available potassium (K) levels in the soil after harvest are presented in Table 11, which showcases the results of different farming methods and genotypes. The findings suggest that the farming techniques employed significantly influenced the K content available in the soil. The study found that the greatest concentrations of accessible K were seen in fields with reduced tillage practices, followed by those with zero tillage and conventional tillage methods. The observed occurrence can be ascribed to the mineralization of potassium, a process that is promoted by the inclusion and subsequent decomposition of agricultural leftovers and fertilizers. The presence of an excessive amount of K in zero tillage can be attributed to a greater retention of K in a condition of equilibrium. This equilibrium state is maintained by the desorption of K from its fixed form within the soil and clay micelles, leading to replenishment of K in the soil solution. This discovery is consistent with the investigation carried out by Dorneles et al. [57], which also documented higher levels of accessible K in zero tillage systems as opposed to conventional tillage systems. The natural farming approach exhibited a notable decrease in the availability of K. Conversely, the genotypes of wheat and rice exhibited no substantial influence on the soil’s accessible K concentration.

The findings derived from the data indicate that the levels of soil organic carbon following the harvest of rice and wheat demonstrate that the impact of cultivation methods and various genotypes on soil organic carbon availability is not statistically significant, with the exception of cultivation methods during the rainy season of 2020, winter season of 2020-21, and rainy season of 2021 (as presented in Table 11). The study observed a notable increase in soil organic carbon content in the reduced tillage condition, followed by the zero-tillage treatment. Conversely, the conventional tillage treatment exhibited a significantly lower soil organic carbon content. The findings exhibited a resemblance to the study conducted by Bhatt [56], whereby it was observed that tillage practices did not exert a substantial impact on the soil organic matter content in clay loam soils during the initial stages.

The observed rise in soil organic carbon resulting from reduced tillage practices may be attributed to an enhanced decomposition of agricultural leftovers, which is strongly correlated with an increase in soil organic carbon content. The practice of incorporating crop residue from the previous crop into the soil by tillage, coupled with efforts to retain the soil, has been found to enhance soil aeration and stimulate microbial activity. Consequently, this process facilitates the mineralization of soil organic matter. The practice of straw retention is effective in maintaining optimal ratios of carbon to nitrogen (C: N) and levels of soil organic matter, hence leading to an increase in organic carbon content. Similar finding indicating significantly higher values of soil organic carbon under reduced tillage have also been reported by other workers [55, 58]. Based on the findings of Bhattacharya et al. [59], it has been observed that the implementation of short-term conservation tillage practices leads to the accumulation of carbon in the uppermost layer of soil. The soil organic carbon content seen in the traditional treatment was found to be the lowest. This could perhaps be attributed to the practice of ploughing, which introduces air into the soil and promotes the oxidation of organic matter present in the soil.

After conducting tests on various kinds, it was found that none of the treatments had a substantial impact on soil organic carbon levels following each season. The variety V_2_ exhibited the highest levels of soil organic carbon in both wheat (HPW 368) and rice (Him Palam Dhan 1) during the duration of the study. Conversely, the variety V1 displayed the lowest levels of soil organic carbon in both wheat (HPW 349) and rice (Sukara Dhan 1). This phenomenon could perhaps be attributed to the improved dispersion of water and essential nutrients throughout the soil profile, as well as the utilization of these elements by a wide range of species, leading to enhanced cycling of accessible resources.

Regarding all the factors described above, including yield attributes, yield, nutrient content, and the availability of nitrogen (N), phosphorus (P), potassium (K), and soil organic carbon, pertaining to the cultivation of wheat and rice crops. The interaction between the soil cultivation methods and the genotypes was found to be non-significant.

## Conclusion

This study offers persuasive proof of the significant effects of different cultivation techniques on soil characteristics and crop production components. The use of conventional tillage, which consistently outperforms natural farming across all four cultivation approaches in terms of yield-contributing factors and overall productivity, has emerged as the most efficient method for cultivating wheat and rice crops. Furthermore, natural farming has produced the lowest levels of equivalent nutrients, whereas conventional tillage has been linked to higher levels of important nutrients in both the grain and straw of these crops, increasing nutrient uptake. In contrast, soils treated with reduced tillage have higher amounts of soil organic carbon (SOC), nitrogen (N), phosphorus (P), and potassium (K). Specific wheat genotype HPW 368 and rice genotypes, particularly Him Palam Lal Dhan 1, have shown higher performance throughout this experiment in terms of yield-contributing variables and total productivity when grown under direct-seeded upland circumstances. Therefore, in the context of the conventional wheat-rice cropping system, this study emphasizes the potential advantages of implementing reduced tillage practices and residue management.

## Data Availability

Data will be made available on request.
